# Opicapone in Parkinson's Disease on Levodopa‐Carbidopa Intestinal Gel Treatment: A Pilot, Randomized Study

**DOI:** 10.1002/mdc3.70231

**Published:** 2025-07-15

**Authors:** Fabiana Colucci, Andrea Gozzi, Pietro Antenucci, Ilaria Casetta, Lorenza Maistrello, Annibale Antonioni, Emanuela Maria Raho, Ginevra Tecilla, Jay Guido Capone, Mariachiara Sensi

**Affiliations:** ^1^ Università degli Studi di Ferrara Ferrara Italy; ^2^ Parkinson and Movement Disorders Unit, Department of Clinical Neurosciences Fondazione IRCCS Istituto Neurologico Carlo Besta Milan Italy; ^3^ Department of Neuroscience and Rehabilitation Azienda Ospedaliero‐Universitaria S. Anna Ferrara Italy; ^4^ IRCCS San Camillo Hospital Venice Italy; ^5^ APSS Ospedale S. Maria del Carmine Rovereto Italy

**Keywords:** Parkinson's disease, levodopa/carbidopa intestinal gel, Opicapone, homocysteinemia, dyskinesias

## Abstract

**Background:**

Levodopa‐carbidopa intestinal gel infusion (LCIG) is an effective therapy for advanced Parkinson's disease (PD). Opicapone (OPC) is an enzyme inhibitor that enhances the bioavailability of levodopa in the brain.

**Objectives:**

This study evaluates the effect of Opicapone addition in PD‐LCIG patients, assessing its impact on motor fluctuations and dyskinesias. Secondly, the study analyses the impact of OPC on non‐motor symptoms, LCIG dosage, and peripheral neuropathy.

**Methods:**

In this pilot study, 22 PD patients on LCIG were randomized to receive OPC or not, based on persistent or reemergent fluctuations. The Movement Disorder Society Unified Parkinson's Disease Rating Scale (MDS‐UPDRS), Unified Dyskinesia Rating Scale (UDysRS), Montreal Cognitive Assessment (MoCA), electroneurography (ENG), LCIG doses, homocysteine, vitamin B12, and folic acid levels were measured at baseline (T0) and after 12 months (T1).

**Results:**

Eleven patients added OPC (addOPC group), while 11 maintained standard treatment (nOPC group). At baseline, both groups had similar disease duration and severity. At T1, the addOPC group showed significant: (i) improvement in motor fluctuations evaluated by the MDS‐UPDRS part IV; (ii) reduction in dyskinesias (UDyRS); (iii) decrease in LCIG infusion rate; (iv) improvement in motor and non‐motor symptoms (MDS‐UPDRS parts I‐III); (v) increase in Vitamin B12. No significant differences were observed in the ENG data, and no serious adverse events occurred. Four addOPC patients (36%) discontinued OPC after 15 ± 2 months, mainly due to hallucinations.

**Conclusions:**

OPC addition appeared well tolerated and beneficial in reducing motor fluctuations, dyskinesia, and LCIG dose. Randomized controlled trials are needed to confirm these findings.

In the advanced stage of Parkinson's Disease (PD), after 5–10 years of illness, the development of motor fluctuations associated with dyskinesias and axial symptoms reduces quality of life and increases disability.^1^, ^2^

Levodopa‐carbidopa intestinal gel (LCIG) is an established therapy for advanced PD in cases of motor fluctuations.^3^, ^4^ Properly selecting patients for this device‐aided therapy has become crucial, considering the need to maintain sustained long‐term control of motor fluctuations and concomitant efficacy without adverse effects on non‐motor symptoms.^5^, ^6^ While a clear benefit on motor fluctuations is reported in the first years of LCIG treatment,^7^, ^8^ a percentage of patients report an unsatisfactory response to LCIG, characterized by a narrow therapeutic window with persistence of off periods, mostly nocturnal, early morning, and/or post‐meal afternoon,^8^, ^9^, ^10^ associated with disabling dyskinesias, sometimes of a complex type.^1^, ^2^ In some cases, attempts to correct this condition have led clinicians to over‐ or under‐modulate the continuous levodopa infusion, resulting in the appearance of motor symptoms (disabling dyskinesias) and non‐motor behavioral side effects (confusion, hallucinations, agitation) or the reappearance of motor‐off periods during the day, ultimately recreating a pulsatile dopaminergic stimulation.

In these cases, theoretically, the addition of Catechol‐O‐methyltransferase inhibitors (COMT‐Is), such as entacapone, opicapone and tolcapone, by reducing the peripheral metabolism of levodopa,^5^, ^11^, ^12^ and increasing its bioavailability in the brain, may optimize the pharmacokinetics of LCIG, ameliorating diurnal and nocturnal motor fluctuations not controlled by LCIG infusion while decreasing the total Levodopa (LD) requirement.

Furthermore, reducing the total amount of LCIG administered to the patient could help lower the costs associated with LCIG treatment,^13^ and mitigate LCIG‐induced hypovitaminosis, potentially preventing the onset of LCIG‐related polyneuropathy,^14^ or at least improving the management of its progression.^15^

Even though COMT‐Is are considered first‐line support in the complex phase of PD, the literature contains limited data on the addition of COMT‐Is in LCIG patients, mostly referring to entacapone, a short‐acting COMT‐I.^16^, ^17^

Opicapone (OPC), a third‐generation, long‐acting COMT‐I, is effective in reducing motor fluctuations induced by the end‐dose effect of oral LD due to its inhibition of peripheral LD metabolism, thereby increasing its bioavailability centrally.^18^ Additionally, unlike Entacapone and Tolcapone, OPC has a longer half‐life and provides a sustained COMT inhibition, offering the advantage of one‐daily oral administration.^18^, ^19^ In addition, OPC does not require the implantation of a new infusion system,^16^ and appears more cost‐effective.^13^ OPC has also been shown to improve vitamin B group absorption and influence homocysteine levels.^20^ In PD patients on LCIG, OPC has been shown to ameliorate vitamin B6 levels.^21^ Based on prior knowledge, the primary objective of the study is to evaluate the effect of OPC on motor fluctuations and dyskinesias in patients with PD on LCIG therapy. The secondary aim is to assess the effect of OPC on motor, non‐motor symptoms, LCIG dosing, and on the prevention or modulation of peripheral neuropathy, vitamin B and homocysteine (Hcy) plasma levels.

## Methods

### Study Design

This pilot, prospective, randomized, open‐label, blinded end‐point study^22^ was conducted at the Movement Disorder Centre of Ferrara Hospital. The study protocol received approval from the local ethics committee (CE‐AVEC 480/2022/Oss/AOUFe) and has been registered on ClinicalTrials.gov (ID: NCT06432309).

### Participants and Recruitment

Between July 2022 and December 2023, we enrolled 22 consecutive Parkinson's disease (PD) patients, diagnosed according to the Movement Disorder Society criteria,^23^ who were already receiving intrajejunal infusion of levodopa‐carbidopa intestinal gel (LCIG). PEG‐J dislocation or malfunctioning as a possible cause of residual or re‐emergent fluctuations was ruled out in all patients prior to study enrollment. Inclusion criteria were: (i) LCIG implantation within 30 months prior to study enrollment; (ii) presence of nocturnal akinesia [assessed via medical history and item 2.9 of the Movement Disorder Society‐Unified Parkinson's Disease Rating Scale (MDS‐UPDRS) part II (≥1)]; (iii) and/or persistence of morning or afternoon akinesia [assessed through item 4.3 in MDS‐UPDRS part IV (≥1)].

Exclusion criteria included: (i) patients with Hoehn & Yahr (H&Y) >4, (ii) LCIG implantation more than 30 months prior to enrollment, (iii) a diagnosis of PD dementia (as defined by MDS criteria^24^), (iv) or non‐compliance with treatment and follow‐up visits.

### Randomization and Intervention

Enrolled patients were randomly assigned in a 1:1 ratio to either receive OPC or continue LCIG without OPC using a web‐based randomization system (National Cancer Institute Clinical Trial Randomization Tool). The randomization list was generated by computer, stratified according to disease duration and severity (H&Y stage). Raters were blinded to treatment assignment.

### Setting and Study Procedure

All study visits were conducted by expert neurologists at the Movement Disorder Centre of S. Anna University Hospital in Ferrara. Blood collection and instrumental assessments were carried out at the site center.

All outcome measures were evaluated at baseline (T0) and at 12 months (T1). During each visit, a neurological motor assessment was conducted using the MDS‐UPDRS III and IV, Unified Dyskinesia Rating Scale (UDysRS), and the Montreal Cognitive Assessment (MoCA). Additionally, data regarding demographic and clinical data were collected: age, sex, age at disease onset and duration, months since LCIG implantation, and PD and non‐PD therapies.

LCIG dosage and concomitant medications were reviewed at each visit to calculate the levodopa equivalent daily dose (LEDD). In accordance with the available literature, the LEDD for OPC was calculated as 50% of the LEDD of levodopa.^11^, ^25^ Throughout the observation period, changes in oral antiparkinsonian therapies and other treatments were not allowed. No patient or caregiver was permitted to modify the LCIG flow rate independently. Extra doses were permitted. In cases where ≥3 extra doses per day were required, an increase in the LCIG continuous dose was considered for all patients. At the same time points (baseline and T1), patients and/or caregivers completed MDS‐UPDRS parts I and II. Points from specific MDS‐UPDRS part III sub‐items were summed to assess rigidity (3.3a to 3.3e), bradykinesia (3.4a to 3.8b, 3.14), and tremor (3.15a, to 3.17e, 3.18). Furthermore, they filled out questionnaires regarding the effect of on‐dyskinesias on daily activities, as assessed in UDysRS Part 1B, and off‐dystonia, as evaluated in UDysRS Part 2B. Intermediate visits were conducted as needed in both groups to adjust the LCIG infusion rate in case of loss of efficacy on motor fluctuations and/or disabling dyskinesias.

Safety data (adverse events and dropouts) were recorded for an additional 6 months after T1.

### Biochemical and Instrumental Assessments

At T0 and T1, blood levels of vitamin B12, folic acid, and homocysteine were measured in the same laboratory. All patients were on a standardized daily oral dose of Vitamin B complex supplement, consisting of 5 μg/day of vitamin B12, 400 μg/day of folic acid, 3.0 mg/day of vitamin B6 and 2.4 mg/day of vitamin B2 for 10 days every month,^26^ starting from the beginning of LCIG treatment and continuing throughout the follow‐up period. Electroneurography of the lower limbs was performed in the same laboratory, calculating the sensory action potential (SAP) of the sural nerve bilaterally. Polyneuropathy was defined as the presence of sural SAPs with an amplitude <6 μV (normative standards of the electromyography laboratory at Sant'Anna University Hospital).

### Data Collection

The data collection form was created using Excel. Cases were identified with alphanumeric codes, and the database was accessible only to researchers during this phase. The Excel file was password‐protected.

### Outcome Measures

The primary outcome measure was the change in motor fluctuations and dyskinesias, assessed using the total MDS‐UPDRS part IV and UDysRS, from the initial assessment to the 12‐month follow‐up.

Secondary outcomes included changes in MDS‐UPDRS part I, part II, and part III, LCIG dosage, sural nerve SAP amplitude in electroneurography, and levels of vitamin B12, folic acid, and homocysteine between the two assessment visits.

In addition, we conducted a spin‐off analysis based on the timing of OPC addition relative to LCIG duration in addOPC group to assess whether an earlier initiation of OPC in patients receiving LCIG influenced the outcome.

### Statistical Analysis

Patient characteristics were summarized using standard descriptive statistics. Categorical variables were reported as frequencies (*n*) and percentages (%), while continuous variables were presented as mean and standard deviation (± SD), or as median and interquartile range (IQR), as appropriate.

Baseline differences in patient characteristics between the two groups at timepoint T0 were assessed using appropriate statistical tests. Categorical variables (eg, sex) were analyzed using Pearson's Chi‐square test or Fisher's exact test, as appropriate. Continuous variables were compared using either the parametric Student's *t*‐test or the non‐parametric Mann–Whitney *U* test, depending on the distribution, which was assessed using the Shapiro–Wilk test.

Changes in motor fluctuations and dyskinesias from baseline (T0) to the 12‐month follow‐up (T1) were analyzed within each group. To assess significant within‐group differences over time, either the paired Student's *t*‐test or the Wilcoxon signed‐rank test was used, depending on the normality of the data.

To evaluate between‐group differences in treatment effects, the changes in outcome measures (ΔT1–T0) were compared using the unpaired Student's *t*‐test or the Mann–Whitney *U* test, as appropriate.

Due to the small sample size, Hedge's g effect sizes, along with their 95% confidence intervals (CIs), were calculated for statistically significant comparisons. Significance was set at *P* < 0.05. Statistical analyses were performed with SPSS Statistics (Version 25).

## Results

A total of 22 PD patients treated with LCIG were enrolled in the study. Of these, 11 (50%) were randomly assigned to receive OPC in addition to LCIG (addOPC group) and 11 continued LCIG without OPC (nOPC group).

Twelve males and 10 females were recruited. The median age was 70 (IQR: 9) years, and the prevalent PD phenotype was tremor‐dominant (68.2%). The average MoCA score was 24.91 ± 4.16.

Table 1 summarizes baseline demographics and PD‐related characteristics of all 22 patients, divided into the two groups: 11 in the nOPC group and 11 in the addOPC group (prior to the addition of OPC). All patients presented nocturnal akinesia, early morning OFF periods, and post‐meal off episodes. Moreover, 7 out of 11 (63.6%) patients in each group presented with disabling dyskinesias. Both groups were similar at baseline in terms of disease duration, severity (H&Y stage), cognitive status, and total LEDD. Patients in the addOPC group were younger than those in the nOPC group at the time of LCIG implantation (*P* = 0.012). Additionally, the addOPC group exhibited higher scores at MDS‐UPDRS Part I (*P* = 0.047) and part IV (*P* = 0.013).

**TABLE 1:** Demographics and Parkinson's disease‐related data at baseline (T0)

Variables	nOPC (*n* = 11)	addOPC (*n* = 11)	*p*‐value
Sex (M/F)	4 (36%)/7 (64%)	8 (73%)/3 (27%)	0.087
H&Y	3.0 (±0.2)	2.8 (±0.6)	0.285
Age at PD onset (years)	57.7 (±6.7)	51.0 (±8.7)	0.085
PD subtype			1
Akineto‐rigid	3 (27.3%)	3 (27.3%)	
Tremor‐dominant	6 (54.5%)	8 (72.7%)	
Mixed	1 (9.1%)	0 (0.0%)	
Disease duration (years)	14.6 (±5.7)	16.2 (±9.5)	0.356
Age at LCIG Implant (years)	71.6 (5.8)	66.1 (4.5)	0.012*
LCIG treatment (years)	1.1 (±0.7)	0.9 (±0.8)	0.085
LEDD	1053.2 (±306.0)	1327.3 (±533.2)	0.07
Total (mg)	954.5 (327.1)	1026.0 (383.8)	0.949
LCIG (mg)	27.3 (±37.8)	40.9 (±53.0)	0.488
Controlled‐release Levodopa (mg)	53.2 (±95.3)	130.9 (±152.1)	0.194
Dopamine‐agonists (mg)	0.0 (±0.0)	29.5 (±52.7)	0.078
MAO‐Is (mg)	18.2 (±60.3)	100.0 (±134.2)	0.071
Amantadine (mg)			
Levodopa‐Carbidopa Intestinal gel	7.0 (±2.2)	8.6 (±2.9)	0.67
Morning dose (mL)	3.0 (±0.9)	2.8 (±0.8)	0.48
Infusion rate (mL/hour)			
Montreal Cognitive Assessment	25.9 (±2.3)	23.9 (±5.3)	0.392
MDS‐UPDRS I	10.1 (±5.0)	14.3 (±4.1)	0.047*
MDS‐UPDRS II	16.8 (±6.6)	21.6 (±9.9)	0.263
MDS‐UPDRS III	37.2 (±8.4)	45.1 (±19.6)	0.669
MDS‐UPDRS IV	6.9 (±1.8)	9.5 (±2.0)	0.013*
UDysRS	16.5 (±12.8)	25.2 (±11.4)	0.292
Polyneuropathy (n)	4 (36.3%)	4 (36.3%)	1000

*Note*: Data are expressed as mean (±standard deviation) for continuous variables and absolute frequencies and percentages (%) for categorical variables.

Abbreviations: addOPC, patients' group with OPC; F, female; H&Y, Hoehn and Yahr; LCIG, Levodopa‐Carbidopa Intestinal gel; LEDD, Levodopa Equivalent Daily Dose; M, male; MAO‐Is, Monoamine Oxidase Inhibitors; MDS‐UPDRS, Movement Disorder Society–Unified Parkinson's Disease Rating Scale; nOPC, patients’ group without OPC; OPC, Opicapone; PD, Parkinson's Disease; UDysRS, Unified Dyskinesia Rating Scale.

At the time of enrollment, four patients (36.4%) in each group (nOPC and addOPC) had a history of polyneuropathy (PN) (Table 1).

At baseline and at the 12‐month follow‐up, 4/11 (36.4%) patients in the nOPC group were on dopamine agonists (DA), and 4/11 (36.4%) were on controlled‐release LD before bedtime. In the addOPC group, 7/11 (63.6%) patients were on DA, and 5/11 (45.4%) were on controlled‐release LD. At baseline, 3/11 (27.3%) of the addOPC group were on Monoamine Oxidase inhibitors (MAO‐Is), compared to none in the nOPC group. Additionally, 5/11 (45.4%) of the addOPC were on amantadine, whereas only 1/11 (9.1%) of the nOPC was taking this treatment. The mean LEDD of oral antiparkinsonian therapy was 303.7 ± 274.9 mg and 98.6 ± 117.7 mg in the addOPC group and in the nOPC group, respectively (*P* = 0.057). The number of extra doses used was similar between groups, with a mean of 0.9 ± 0.6 per day per patient in the addOPC group and 1.2 ± 0.7 in the nOPC group (*P* > 0.05). Intermediate visits required for therapy adjustments were similar in both groups (3.8 ± 1.1 in nOPC group vs. 4.0 ± 0.9 in the addOPC group, *P* > 0.05).

Patients in the addOPC group showed significant improvements in motor fluctuations and dyskinesias assessed by MDS‐UPDRS Part IV (*P* < 0.05) and UDysRS total score (*P* < 0.05) compared to the nOPC group. A significant improvement was also observed in MDS‐UPDRS Part III (*P* < 0.01). Analyses of MDS‐UPDRS III compound sub‐items revealed improvements in rigidity (*P* < 0.005) and bradykinesia (*P* < 0.005) in the addOPC group compared to nOPC group, though no significant improvement was observed in tremor. As for non‐motor symptoms, the addOPC group demonstrated significant improvements in MDS‐UPDRS Parts I and II (*P* < 0.05). No significant difference between the two groups in the change in MoCA scores from baseline was observed (Table 2).

**TABLE 2:** The mean difference T1‐T0 between PD‐LCIG patients without Opicapone (nOPC) and those with Opicapone (add‐OPC)

Variables	ΔnOPC (*N* = 11)	ΔaddOPC (*N* = 11)	*P*‐value	Effect size (95% CI)
LCIG morning dose	0.9 (±1.8)	−0.4 (±1.0)	0.252	
LCIG continuous dose	0.1 (±0.6)	−0.4 (±0.4)	<0.05*	**0.95 (0.09–1.8)**
Total LEDD	53.7 (±164.3)	76.6 (±444.6)	0.871	
LEDD LCIG	61.7 (±100.3)	−49.3 (±305.5)	<0.005*	**0.48 (0.46–1.28)**
MDS‐UPDRS				
Part I	2.5 (±3.0)	−3.1 (±3.9)	<0.005*	**1.54 (0.59–2.46)**
Part II	2.3 (±2.4)	−5.4 (±5.6)	<0.005*	**1.71 (0.74–2.66)**
Part III (total)	4.0 (±6.9)	−10.0 (±11.7)	<0.001*	**1.4 (0.47–2.30)**
Part III (rigidity)	0.6 (±1.6)	−2.0 (±2.6)	<0.005*	**1.17 (0.27–2.03)**
Part III (bradikynesia)	1.7 (±3.1)	−4.5 (±3.6)	<0.005*	**1.77 (0.78–2.73)**
Part III (tremor)	0.1 (±1.2)	−1.1 (±1.9)	0.100	
Part IV	0.2 (±1.0)	−2.3 (±3.1)	<0.005*	**1.03 (0.15–1.8)**
UDysRS	0.6 (±4.0)	−5.1 (±10.0)	0.046*	**0.72 (0.12–1.61)**
Sural SAP amplitude (μV)				
Left	−2.0 (±4.5)	−1.0 (±2.1)	0.534	
Right	−2.1 (±3.6)	−0.8 (±1.7)	0.281	
Peroneal cMAP (mV)				
Left	−0.2 (±2.4)	−0.2 (±1.7)	0.963	
Right	0.3 (±1.7)	0.3 (±1.7)	0.985	
MoCA	−2.3 (±2.1)	−1.0 (±1.0)	0.064	

*Note*: Data are expressed as mean (standard deviation) for continuous variables and number for categorical variables.

Abbreviations: CI, Confidence Interval; cMAP, compound Motor Action Potential; LCIG, Levodopa‐Carbidopa Intestinal gel; LEDD, Levodopa Equivalent Daily Dose; MoCA, Montreal Cognitive Assessment; MDS‐UPDRS, Movement Disorder Society–Unified Parkinson's Disease Rating Scale; PD, Parkinson's Disease; SAP, Sensory Action Potential; UDysRS, Unified Dyskinesia Rating Scale.

**Figure 1:**
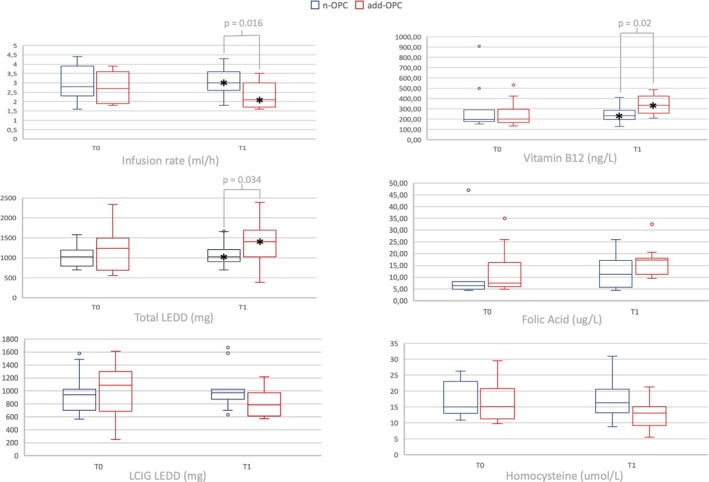
Median values at T0 and T1 for levodopa/carbidopa intestinal gel infusion rate (LCIG), total and LCIG levodopa equivalent daily dose (LEDD), vitamin B12, homocysteine and folic acid, comparing LCIG therapy alone (n‐OPC) with opicapone association (add‐OPC). *: significant differences.

At the 12‐month follow‐up (T1), patients in the addOPC group showed a significant reduction in both LCIG dosage (mean change in LEDD −49.3 mg in addOPC vs. +61.7 mg in nOPC, *P* < 0.05) and continuous infusion rate (mean change −0.40 ml/h in addOPC vs. +0.1 ml/h in nOPC, *P* < 0.05). The median infusion rate was also lower in the addOPC group (2.1 ml/h in addOPC vs. 3.0 ml/h in nOPC, *P* = 0.016) compared to baseline. Total LEDD at T1 resulted higher the addOPC group compared to nOPC group (median total LEDD of 1404.0 in add OPC vs 1026.0 in nOPC; *P* = 0.034) (Fig. 1 and Table 2).

Electroneurographic parameters did not differ significantly between groups, and there was no new onset or worsening of pre‐existing PN (Table 2).

In terms of laboratory analyses, we observed a significant increase in Vitamin B12 levels at T1 in the addOPC group (mean values 247.1 ± 80.2 ng/L in nOPC vs. 348.9 ± 93.4 ng/L in addOPC, p = 0.015; mean difference T1‐T0–57.3 ± 235.1 ng/L in nOPC vs. 92.2 ± 112.6 ng/L in addOPC, p = 0.02), even though the standardized dose of the Vitamin B complex supplement remained unchanged in all 22 patients. Additionally, while the homocysteine levels in the addOPC group decreased compared to nOPC group (nOPC: 17.6 ± 6.4 μmol/L vs. addOPC: 12.7 ± 4.7 μmol/L) approaching statistical significance (*P* = 0.053), no significant change was observed in folic acid levels between the groups (Fig. 1 and Table S1).

Seven patients in the addOPC group had been on LCIG for a variable period (mean of 16 ± 8 months) prior to adding OPC (late OPC addition, L‐OPC group), while four patients added OPC within 45 ± 10 days of PEG‐J positioning (early OPC addition, E‐OPC). At baseline, patients in the E‐OPC group had significantly higher scores on MDS‐UPDRS Part III (L‐OPC: mean 42.1 ± 15.7 vs. E‐OPC: mean 50.25 ± 27.0, *P* < 0.05), although their disease duration was shorter (L‐OPC: 23.7 ± 10.4 years vs. E‐OPC 14.0 ± 2.8 years, *P* < 0.05). No other significant differences were observed between the groups in terms of age, sex, age at PD onset, total and LCIG LEDD, age of LCIG implantation, or MDS‐UPDRS Parts I, II, and IV or UDysRS total score. At T1, the E‐OPC group showed a significant reduction in UPDRS Part IV total score (L‐OPC: −0.71 ± 2.7 vs. E‐OPC: −5 ± 1.1, *P* = 0.042), as well as in the sub‐item 4.3 (time spent in OFF periods) of MDS‐UPDRS Part IV (L‐OPC: −0.3 ± 0.5 VS. E‐OPC: −1.25 ± 0.5, *P* = 0.042) respect to L‐OPC group. No other statistically significant differences were found during the observation period.

All the patients were monitored for an additional 6 months after T1 for safety assessment. No serious adverse events related to the devices or treatments (OPC and/or LCIG) were reported, and no patients discontinued treatment during the 12‐month observation period. In the nOPC group, three patients discontinued LCIG after 13, 15 and 14 months due to harmful hallucinations, lack of a caregiver, repeated pulling of PEG‐J, and nighttime confusion episodes in the onset of cognitive decline, respectively. In contrast, no patients in the addOPC group discontinued LCIG. However, four patients (36%) in the addOPC group discontinued OPC after 15 ± 2 months due to the onset of hallucinations and nighttime confusion. All four patients who discontinued OPC were from the L‐OPC group. At baseline, they had a longer disease duration, lower MDS‐UPDRS Part IV total score, and lower MoCA score compared to patients who continued OPC treatment (see Table 3 and Table S2).

**TABLE 3:** Baseline data in addOPC divided according to discontinuation of OPC

Variables	Ongoing‐OPC (*n* = 7)	Drop‐out OPC (*n* = 4)	*p*‐value
Age (years)	67.0 (±4.2)	72.0 (±4.1)	0.072
Disease duration (years)	12.4 (±3.4)	22.5 (±11.1)	<0.05*
LEDD T0	1469.0 (±475.7)	1159.5 (±643.4)	0.382
LCIG Morning dose	9.5 (±2.6)	7.0 (±3.1)	0.191
LCIG Continuous dose	2.8 (±0.8)	2.7 (±1.0)	0.744
MDS‐UPDRS I	14.9 (±4.7)	13.3 (±3.3)	0.565
MDS‐UPDRS II	21.4 (±11.5)	22.0 (±7.7)	0.932
MDS‐UPDRS III	49.4 (±23.5)	37.5 (±7.7)	0.358
MDS‐ UPDRS IV	10.6 (±1.4)	7.5 (±1.3)	<0.01*
UDysRS	25.6 (±14.2)	24.5 (±4.0)	0.890
MoCA	26.7 (±3.5)	19.0 (±4.6)	**<0.05**

*Note*: Data are expressed as mean (± standard deviation).

Abbreviations: LCIG, Levodopa‐Carbidopa Intestinal gel; LEDD, Levodopa Equivalent Daily Dose; MDS‐UPDRS, Movement Disorder Society–Unified Parkinson's Disease Rating Scale; MoCA, Montreal Cognitive Assessment; OPC, Opicapone; UDysRS, Unified Dyskinesia Rating Scale.

## Discussion

This pilot study explored the effects of OPC addition in a group of PD patients treated with LCIG. Eleven patients receiving LCIG without OPC addition were compared with 11 patients receiving LCIG combined with OPC. At baseline all patients experienced nocturnal akinesia, early morning off, and post‐meal afternoon off episodes, despite prior adjustments to the LCIG infusion rate.

The challenge of achieving true continuous dopaminergic stimulation (CDS) in PD patients, even with continuous drug delivery (CDD) via therapies such LCIG, is well‐documented.^6^, ^27^ This issue is multifactorial, involving peripheral factors (eg, constipation, Helicobacter pylori infection, small intestinal bacterial overgrowth, protein‐rich meals), central factors (eg, loss of long‐duration response, reduced brain penetration, and the involvement of non‐dopaminergic pathways) and device‐related issues (pump failure, line blockage or tube dislocation).^28^

Additionally, LCIG therapy increases plasma levels of 3‐O‐methyldopa (3‐OMD), a metabolite of levodopa that competes with levodopa for brain uptake.^29^ This phenomenon could be reduced by combining a COMT inhibitor.^10^, ^30^

The proof‐of‐concept for the efficacy and safety of adding a COMT‐I to LCIG therapy was established in a short‐term pilot study by Nyholm et al.,^31^ who demonstrated that administering Entacapone or Tolcapone at 5‐hour intervals reduced LCIG dosages by 20%. Subsequent studies confirmed these findings, leading to the approval of LCIG combined with entacapone (LECIG) in several European countries since 2018.^32^, ^33^, ^34^

No data on the efficacy and safety of OPC as add‐on therapy in patients receiving LCIG are currently available in the literature. Nevertheless, OPC is frequently used in clinical practice as a therapeutic strategy.^35^, ^36^ It represents a valuable alternative to LECIG, particularly in countries where LECIG is not available. OPC offers several potential advantages, including easy administration, the possibility of discontinuation without need to interrupt LCIG treatment, and favorable cost‐effectiveness. In this regard, Leta and colleagues reported a 24.8% reduction in daily LCIG dose in 11 patients receiving OPC add‐on therapy, resulting in a significant reduction in treatment costs.^13^

Moreover, no studies have yet analyzed non‐motor symptom outcomes or dropout causes.

In our study, at baseline the two groups were comparable regarding disease duration, Hoehn and Yahr staging, cognitive status (within normal limits) and total LEDD but add‐on OPC presented younger age and more severe fluctuations in respect to nOPC group. Mild polyneuropathy (PN) was present in 4/11 in both groups.

At 12 months of follow‐up, the OPC group demonstrated a clinically meaningful improvement in motor complications (MDS‐UPDRS Part IV) and Dyskinesia scores (UDysRS). Motor symptoms (MDS‐UPDRS Part III), non‐motor symptoms, assessed by MDS‐UPDRS Parts I and II, improved as well (Table 2), without worsening of cognitive outcomes.

OPC add‐on didn't significantly influence LCIG‐related PN nor exerted a protective effect as expected,^21^, ^30^ even though in the OPC group Hcy levels remained stable at follow‐up, and vitamin B12 levels were increased. The relatively short follow‐up period and the chronic use of vitamin B complex supplements in all patients, as per treatment indication reported elsewhere,^26^ could account for the absence of improvement.

The subgroup analysis of the OPC group revealed that early starters (4/11 patients receiving OPC within 1 month of PEG‐J positioning) achieved significant improvements in motor complications (MDS‐UPDRS Part IV), particularly in OFF‐period duration (item 4.3), compared to those with delayed OPC introduction. However, no significant differences in motor and non‐motor outcomes were observed between the early and late OPC subgroups. Although these findings should be interpreted with caution due to the small sample size, they align with recent studies on oral OPC, which suggest that early OPC initiation during the disease course improves motor fluctuations and potentially prevent the emergence of motor complications.^35^, ^36^, ^37^

Adding OPC in patients in the advanced PD with long disease duration may be contraindicated due to potential harmful cognitive effects^35^ and selecting the right patient and the optimal timing can be challenging, particularly in PD patients receiving device‐aided therapies such as LCIG. In this cohort, OPC appears to provide better and more sustained control of motor fluctuations, particularly in patients with shorter disease duration, more disabling motor fluctuations, and less cognitive impairment. Conversely, poorer outcomes seem to be directly associated with longer disease duration. It is conceivable that, in advanced disease stages, CDD may no longer effectively counteract persistent OFF states despite achieving more stable plasma LD levels, likely due to the involvement of non‐dopaminergic mechanisms.^9^

Three out of 11 patients (27%) in nOPC group discontinued LCIG, two of them due to cognitive issues, whereas no LCIG dropouts were recorded in the addOPC group. OPC discontinuation was documented in four patients (36%), all of whom were in late OPC starters. Discontinuation was mainly due to the development of confusion and predominantly nocturnal hallucinations, which warranted cessation of this add‐on therapy. The rate of OPC discontinuation observed in our small case series is notable compared to most recent studies on the safety and efficacy of LECIG, which reported lower discontinuation rates ranging from 1.8% to 13.3%.^33^ Only one study reported a higher dropout rate, with 7 out of 27 patients (26%) discontinuing LECIG after 6 months of follow‐up due to neuropsychiatric issues, narrowed therapeutic window, and disabling dyskinesias. Additionally, three patients were excluded during the nasojejunal tube test phase, two of whom experienced delirium.^38^ Similarly to our data, Leta et al.,^13^ reported that 3 out of 11 patients (27%) discontinued OPC after a shorter follow‐up period (4 months) due to side effects such as hallucinations and lack of efficacy. It is worth noting that, in our four patients, the confusional episodes and hallucinations fully resolved after OPC discontinuation, and continuation of LCIG therapy was still possible in all cases. Possible predictors of OPC discontinuation in these patients included longer disease duration, lower baseline MDS‐UPDRS Part IV scores, and worse cognitive status.

We acknowledge that this pilot trial, presents some limitations inherent to its design, including small sample size, single‐center setting, open‐label nature, and absence of an active comparator or placebo in the control group. However, one advantage of the open‐label PROBE design^22^ is its closer resemblance to routine clinical practice, potentially providing valuable insights applicable to real‐life scenarios. Nevertheless, the presence of potential structural differences between both cohorts at baseline (such as younger age at implantation and more pronounced motor fluctuations in add OPc group) does not permit to generalize these results and the small sample size does not allow for conducting further specific analysis. Therefore, while our findings provide valuable insights, they are preliminary.

We believe that these findings underscore the potential benefits but also the possible harmful adverse events of OPC as an add‐on therapy in LCIG‐treated PD patients, offering clinically relevant insights and serving as a foundation for future larger comparative efficacy and safety studies in this field.

## Author Roles

(1) Research project: A. Conception, B. Organization, C. Execution; (2) Statistical Analysis: A. Design, B. Execution, C. Review and Critique; (3) Manuscript Preparation: A. Writing of the draft, B. Review and Critique. All authors reviewed the manuscript, agreed with its content, and approved its submission. All the authorship criteria have been met.

F.C.: 1B, 1C, 3A, 3B;

A.G.: 1A, 1C, 3A, 3B;

P.A.: 2A, 2B;

I.C.: 2A, 2B, 2C;

L.M.: 2C; AA: 1C;

E.M.R.: 1C;

G.T.: 1C;

J.G.C.: 1C;

M.S.: 1A, 1B, 3A, 3B.

## Disclosures

**Ethical Compliance Statement:** The authors confirm that the approval of an institutional review board was not required for this work. We confirm that we have read the Journal's position on issues involved in ethical publication and affirm that this work is consistent with those guidelines. The manuscript submitted for publication has been performed following ethical standards stated in the 1964 Declaration of Helsinki and its later amendments. Written informed consent was obtained from the patients for the participation in the study and the publication of the data.

**Funding Sources and Conflict of Interest:** Open Access funding was provided by Università degli Studi di Ferrara within the CRUI‐CARE Agreement. The authors declare that there are no conflicts of interest relevant to this work.

**Financial Disclosures for the previous 12 months**: The authors declare that there are no additional disclosures to report.

## Supporting information


**TABLE S1.** Laboratory analyses at baseline and after 12 months after randomization.

**TABLE S2.** Adverse events leading to LCIG or OPC discontinuation observed during the extended observation period (12–18 months after T0) in PD‐LCIG patients treated with LCIH without Opicapone (nOPC) and those with adjunctive Opicapone (addOPC).

## Data Availability

The data that support the findings of this study are available from the corresponding author upon reasonable request.
