# Supplementary material for: Opicapone in Parkinson's Disease on Levodopa‐Carbidopa Intestinal Gel Treatment: A Pilot, Randomized Study
Source: Mov Disord Clin Pract. 2025 Jul 15;12(11):2034–42. doi: 10.1002/mdc3.70231 (PMC12625100; doi:10.1002/mdc3.70231)
Supplement: Supplementary file 1 — TABLE S1. Laboratory analyses at baseline and after 12 months after randomization. [file MDC3-12-2034-s001.docx]

**Table S1.** Laboratory analyses at baseline and after 12 months after randomization.

| ***Variables*** | **Timeline** | **nOPC** | **addOPC** | ***p value*** |
| --- | --- | --- | --- | --- |
| *Homocysteine (umol/l)* | Baseline (T0) | 15.0 (m: 17.6; SD: 5.5) | 13.3 (m: 16.1; SD: 6.2) | *0.955* |
|  | One year (T1) | 16.4 (m: 17.6; SD: 6.4) | 13.1 (m: 12.7; SD: 4.7) | *0.053* |
|  | Δ(T1-T0) | - 0.8 (m: -0.4; SD: 8.7) | - 3.9 (m: -4.5; SD: 5.3) | *0.374* |
| *Vitamin B12 (ng/l)* | Baseline (T0) | 194.0 (m: 290.5; SD:228.0) | 203.0 (m: 256; SD: 122.4) | *0.663* |
|  | One year (T1) | 234.0 (m: 247.1; SD: 80.2) | 333.0 (m: 348.9 SD: 93.4) | ***0.015**** |
|  | Δ(T1-T0) | 13.0 (m: -57.3; SD: 235.1) | 83.0 (m: 92.9 SD: 112.6) | ***0.02**** |
| *Folic Acid (ug/l)* | Baseline (T0) | 6.5 (m: 10.3; SD: 13.0) | 7.5 (m: 12.1; SD: 9.8) | *0.726* |
|  | One year (T1) | 11.1 (m: 12.1; SD: 7.0) | 17.3 (m: 16.7; SD: 6.4) | *0.140* |
|  | Δ(T1-T0) | 5.4 (m: 1.9; SD: 16.7) | 5.2 (m: 4.6; SD: 11,4) | *0.667* |
| The data are presented as medians, with the mean (m) and standard deviation (SD) shown in brackets. Δ(T1-T0): difference between T1 and T0. | | | | |
