# Supplementary material for: Opicapone in Parkinson's Disease on Levodopa‐Carbidopa Intestinal Gel Treatment: A Pilot, Randomized Study
Source: Mov Disord Clin Pract. 2025 Jul 15;12(11):2034–42. doi: 10.1002/mdc3.70231 (PMC12625100; doi:10.1002/mdc3.70231)
Supplement: Supplementary file 2 — TABLE S2. Adverse events leading to LCIG or OPC discontinuation observed during the extended observation period (12–18 months after T0) in PD‐LCIG patients treated with LCIH without Opicapone (nOPC) and those with adjunctive Opicapone (addOPC). [file MDC3-12-2034-s002.docx]

**Table S2.** Adverse events leading to LCIG or OPC discontinuation observed during the extended observation period (12-18 months after T0) in PD-LCIG patients treated with LCIH without Opicapone (nOPC) and those with adjunctive Opicapone (addOPC).

| ***Variables*** | **nOPC (n)** | **AddOPC (n)** |
| --- | --- | --- |
| Hallucinations, n (%) | 1 (9.1%) | 3 (27.3%) |
| Confusion, n (%) | 1 (9.1%) | 2 (18.2%) |
| PEG-J self-removal, n (%) | 1 (9.1%) | 0 (0%) |
| Difficulty in device managing at home, n (%) | 1 (9.1%) | 0 (0%) |
